# Nanofiltration Membranes: Recent Advances and Environmental Applications

**DOI:** 10.3390/membranes12050518

**Published:** 2022-05-13

**Authors:** Mohammad Peydayesh

**Affiliations:** Department of Health Sciences and Technology, ETH Zurich, 8092 Zurich, Switzerland; mohammad.peydayesh@hest.ethz.ch

Nanofiltration (NF) is a cutting-edge filtration technology that may be considered a true paradigm shift in membrane science. NF can be used for a wide range of applications due to its unique properties to address major global issues in a sustainable and green way. Furthermore, NF technology has strong potential for supporting the UN Sustainable Development Goals (SDGs), particularly SDG 6: Clean Water and Sanitation. However, the successful industrial application of NF depends on developing efficient and reliable membranes, optimization of processes, understanding of the separation mechanisms, and justifications in terms of energy and economics.

This Special Issue, titled “Nanofiltration Membranes: Recent Advances and Environmental Applications” in the Membranes, aims to assess the recent developments and advances in all the aspects related to NF technology and its environmental applications.

There are ten contributions, including eight research articles and two reviews, in this Special Issue. Various topics are discussed, including fabrication of organic and inorganic NF membranes, tailoring NF membranes’ surface properties, the application of NF in side-stream valorization, using NF technology in combination with other membrane technologies such as ultrafiltration (UF) and reverse osmosis (RO), wide-range applications of NF for removing organic and inorganic pollutants and viruses, and a detailed sustainability assessment of the use of NF technology via life cycle assessment (LCA).

Higgins et al. [1] studied the removal of Enantiomeric Ibuprofen in an NF membrane process. In addition to the rejection by the NF membrane itself, results show that the stainless steel equipment of a flat-sheet experimental unit could adsorb up to 23% of pharmaceuticals, primarily S-enantiomers of ibuprofen. This illustrates the importance of conducting initial mass balance experiments to determine possible losses of the compound in bench-scale membrane removal studies in order to determine the true removal capabilities of the membrane process.

Zheng et al. [2] reviewed the functions of ionic liquids (ILs) to prepare membranes for different filtration processes, including NF. The application of ILs as raw membrane materials, physical additives, chemical modifiers, and solvents was discussed. Moreover, related challenges and future perspectives of IL-assisted membranes were highlighted.

Colloidal fouling caused by the accumulation of particles such as colloidal silica, iron, aluminum, manganese oxides, and calcium carbonate precipitates on the membrane surface impedes NF operations by introducing additional hydraulic resistance that reduces water permeability. Malakian et al. [3] applied nanoscale line-and-groove patterns on commercial NF membranes using thermal embossing to address this issue. Experimental work combined with CFD simulations revealed that increasing the pattern ratio fraction leads to higher threshold flux, which is vital for the productivity of the NF process since it maintains low fouling rates.

Ainscough et al. [4] evaluated the performance of NF technology for removing chlorinated hydrocarbons such as trichloroethylene (TCE), tetrachloroethylene (PCE), cis- 1,2-dichloroethylene (DCE), 2,2-dichloropropane (DCP), and vinyl chloride (VC) from groundwater. There are few reports on removing these compounds from water, which is considered a particularly difficult separation. The results from the laboratory experiment showed high performance of the NF, where rejection rates of up to 93% for synthetic solutions and up to 100% for real groundwater samples were achieved. In site trials and due to the operational limitation, although the NF removed organic materials, it failed to remove volatile organic compounds (VOCs).

Aziz et al. [5] investigated the performance of UF, NF, and RO technologies for treating the effluent from a full-scale membrane bioreactor (MBR) municipal wastewater treatment plant. While the UF technology showed removal efficiencies of around 40% for chemical oxygen demand (COD), NH_4_^2+^, PO_4_, and NO_3_, NF and RO technologies exhibited excellent removal rates above 90%. The superiority of NF technology was further revealed by the fact that the energy consumption of RO was 1.4 times higher than that of NF.

NF can also be used to valorize industrial by-products, contributing to waste management and the circular economy. Macedo et al. [6] valorized goat cheese whey via an integrated process of UF and NF. The results showed that all the lactose could be retained using NF, allowing total whey recovery in cheese dairy plants.

Mixed matrix membranes (MMMs) are one of the primary membrane categories, combining the advantage of polymeric membranes and inorganic nanoparticles. Siddique et al. [7] reviewed the current state of the art of MMMs in different membrane processes, including NF. The focus here was on different fabrication approaches of MMMs, controlling parameters, fouling mitigation, and challenges and outlooks.

Amadou-Yacouba et al. [8] evaluated the impact of the pre-ozonation step during the NF of MBR effluent. Although the ozonation process had a low effect on the organic carbon mineralization, it decreased the COD and the specific UV absorbance by 50%, indicating the efficiency of ozonation in degrading a specific part of the organic matter fraction. The results showed that pre-ozonation during the use of NF could decrease the fouling and maintain the flux by partially mineralizing dissolved and colloidal organic matter.

Bordbar et al. [9] investigated and benchmarked hybrid NF desalination plants in terms of sustainability and the environmental footprint via LCA in the Persian Gulf region. The studied plants were multi-stage flash (MSF), hybrid RO-MSF, hybrid NF-MSF, RO, and hybrid NF-RO, and their impacts on climate change, ozone depletion, fossil depletion, human toxicity, and marine eutrophication were assessed. The LCA results demonstrated that hybrid NF-RO is a process with minimal environmental impacts for seawater desalination.

NF can be used to remove microorganisms and viruses in water sources prior to the consumption, addressing the problems associated with waterborne diseases. To do so, Yüzbasi et al. [10] developed two ceramic membranes composed of alumina platelets using spray granulation. They assessed the virus retention performance using two virus surrogates: MS2 and fr bacteriophages. The results showed complete virus removal from water, where the virus concentration decreased by 7 log10 reduction value (LRV), meeting the World Health Organization (WHO) criteria of LRV ≥ 4.

In conclusion, I would like to express my sincere appreciation to the authors, reviewers, and publisher for their outstanding work and contribution to the Special Issue “Nanofiltration Membranes: Recent Advances and Environmental Applications”. I hope this collection will be useful for the membrane community worldwide, promoting the NF technology toward a more efficient, sustainable, and affordable process for environmental application.
